# Review of the genus *Stigmus* Panzer (Hymenoptera, Crabronidae) in China, with description of five new species from the Oriental and Palearctic Regions

**DOI:** 10.3897/zookeys.843.31885

**Published:** 2019-05-09

**Authors:** Nawaz Haider Bashir, Qiang Li, Li Ma

**Affiliations:** 1 Department of Entomology, College of Plant Protection, Yunnan Agricultural University, Kunming, Yunnan, 650201, China Yunnan Agricultural University Kunming China

**Keywords:** Hymenoptera, new species, Pemphredoninae, *
Stigmus
*

## Abstract

Five new species of the genus *Stigmus*: *S.capoblongus* Bashir & Ma, **sp. n.**, *S.denticorneus* Bashir & Ma, **sp. n.**, *S.fronticoncavus* Bashir & Ma, **sp. n.**, *S.interruptus* Bashir & Ma, **sp. n.** and *S.lobomelanicus* Bashir & Ma, **sp. n.** are described and illustrated from China. Also, a key to the species of *Stigmus* Panzer occurring in China is provided.

## Introduction

The genus *Stigmus* Panzer was erected by Panzer (1804) on the basis of type species *Stigmuspendulus* Panzer. At present 25 species and 4 subspecies are described worldwide, of which the highest number of species is known from the Nearctic Region (10 species and 2 subspecies), followed by the Palearctic Region (7 species); 4 species and 2 subspecies were found in the Oriental Region (of which 3 species and 2 subspecies were in China), 2 species in Neotropical Region, 1 species in the Palearctic and Oriental Regions, and 1 species in the Nearctic and Neotropical Regions (Morawitz 1864; Tsuneki 1954, 1977; Kolesnikov 1977; Allen 1987; Budrys 1987, 1995; Uffen 1997, 1998; Jones 2001; Pulawski 2018). Recently, *Stigmuseurasiaticus* was well described by Mokrousov from Russia (Mokrousov 2017).

The diagnostic characteristics that differentiate *Stigmus* from other genera in Pemphredonini are the presence of occipital carina; mandibles in the male bidentate, in the female uni-, bi-, or usually tridentate; vertex micropore field (opaque area) present; labrum subtriangular, pentagonal or trapeziform; face with a shallow scapal basin; interantennal tubercle absent; clypeus of male with silvery dense setae; eyes broadly separated, pitted grooves along orbits narrow or absent; head moderately developed behind eyes; pronotum with a transverse carina; notauli indicated or developed; omaulus well developed (only in *S.solskyi* A. Morawitz is it invisible against the background of a wrinkled mesopleuron sculpture); no definitive episternal sulcus; stigma large, two submarginal cells; hindwing media diverging before or beyond cu-a, hindwing median cell normal size; petiole at least twice its diameter; and female pygidial plate present (Valkeila 1956; Krombein 1973; Bohart and Menke 1976; Finnamore 1995).

In the present study, five new species of genus *Stigmus* Panzer are described and illustrated. A key to the species of the genus *Stigmus* reported from China is also provided.

## Materials and methods

The specimens examined in this study belong to the following institutions: Insect Collections of China Agricultural University, Beijing, P. R. China (CAU); Insect Collections of Yunnan Agricultural University, Kunming, Yunnan, P. R. China (YNAU); and Parasitic Hymenoptera Collection of Zhejiang University, Hangzhou, Zhejiang Province, P. R. China (ZJU).

The specimens were observed and illustrated with the help of an Olympus stereomicroscope (SZ Series, Japan) with an ocular micrometer. For the terminology we mainly followed Bohart and Menke (1976). The abbreviations in the text are as follows: BL, body length; HLD, head length in dorsal view (the distance from frons to occipital margin in the middle); HLF, head length in frontal view (the distance from vertex to clypeal margin in the middle); HW, head width (dorsal view, maximum); EW, eye width (lateral view, maximum); EWd, eye width (frontal view, maximum); TW, gena width (lateral view, maximum); EL, eye length (lateral view, maximum); POD, postocellar distance (distance between inner margins of hind ocelli); OOD, ocellocular distance (distance between outer margin of hind ocellus and nearest inner orbit); OCD, ocello-occipital distance (distance between posterior margin of hind ocellus and occipital margin, dorsal view); PW, petiole width (dorsal view, in the middle); PL, petiole length (lateral view); WTI, maximum width of metasomal tergum I (dorsal view); LTI, maximum length of metasomal tergum I (dorsal view).

## Key to the species of *Stigmus* Panzer from China

PR and OR represent Palearctic and Oriental Regions, respectively.

**Females** (unknown for *S.capoblongus* sp. n)

**Table:** 

1	Clypeus deeply impressed, not produced (OR)	***S.fronticoncavus* sp. n.**
–	Clypeus flat or slightly convex, slightly or strongly produced	**2**
2	Scrobal suture inconspicuous, lacking or just a single weak carina (PR)	***S.denticorneus* sp. n.**
–	Scrobal suture narrow or broad, slenderly or distinctly crenate (OR)	**3**
3	Ventral surface of petiole shiny, without carina	***S.kansitakuanus* Tsuneki**
–	Ventral surface of petiole with a few strong longitudinal carinae medially and posteriorly	**4**
4	Ventral gena with large dense punctures mixed with several irregular rugae laterally; lateral surface of petiole with several irregular rugae and two strong lateral carinae medially and posteriorly	***S.lobomelanicus* sp. n.**
–	Ventral gena shiny, smooth; lateral surface of petiole with a few strong longitudinal carinae medially and posteriorly	**5**
5	Pronotal collar with strong lateral carinae, forming round antero-lateral angle; inner orbital furrow broad, shiny, slenderly rugulose	***S.murotai* (Tsuneki)**
–	Pronotal collar without lateral carina or carina incomplete, without antero-lateral angle; inner orbital furrow lacking	**6**
6	Occipital carina complete, distinctly crenulate; scutellum with midsize sparse punctures, median line weakly impressed; posterior surface of propodeum with sturdy reticulation	***S.shirozuialishanus* Tsuneki**
–	Occipital carina incomplete, not crenulate; scutellum with fine sparse punctures, without median line; posterior surface of propodeum with sparse, longitudinal rugae or irregular rugae	**7**
7	Mesoscutum with large punctures, anterior and posterior area with dense longitudinal micro sculptures; vertex with few punctures; pronotal collar with sturdy incomplete anterior carina	***S.interruptus* sp. n.**
–	Mesoscutum with tiny punctures, without micro sculpture or slightly coriaceous anteriorly; vertex impunctate; pronotal collar with strong complete anterior carina	**8**
8	Dorsal surface of petiole distinctly widened toward apex; lateral surface of propodeum with contiguous, slender or sturdy, oblique rugae; admedian and parapsidal line weakly impressed; median and upper frons impunctate; pygidial area impunctate, with dense longitudinal rugae	***S.convergensami* Tsuneki**
–	Dorsal surface of petiole slightly widened toward apex; lateral surface of propodeum reticulate; admedian and parapsidal line distinct; median and upper frons with fine punctures; pygidial area with two lines of large punctures, without rugae	***S.japonicus* Tsuneki**

**Males** (unknown for *S.fronticoncavus* sp. n. and *S.interruptus* sp. n.)

**Table:** 

1	Clypeus nearly flat or flat (OR)	**2**
–	Clypeus reflected toward apex	**3**
2	Frontal furrow lacking; parapsidal line weakly impressed; vertex behind ocelli impunctate	***S.lobomelanicus* sp. n.**
–	Frontal furrow fine and weak on upper frons, anteriorly deeper, broader and distinct; parapsidal line distinct; vertex behind ocelli with fine punctures	***S.kansitakuanus* Tsuneki**
3	Scrobal suture inconspicuous, lacking, or just a single weak carina (PR)	***S.denticorneus* sp. n**
–	Scrobal suture broad, distinctly crenate and complete (OR)	**4**
4	Pronotal collar with strong lateral carinae, forming round antero-lateral angle	***S.murotai* (Tsuneki)**
–	Pronotal collar without lateral carina, without antero-lateral angle	**5**
5	Median and upper frons with punctures; vertex behind ocelli with punctures; propodeal enclosure U-shaped medially; dorsal surface of petiole slightly widened toward apex	**6**
–	Median and upper frons impunctate; vertex behind ocelli impunctate; propodeal enclosure triangular medially; dorsal surface of petiole distinctly widened toward apex	**7**
6	Frontal furrow weakly impressed; gena with fine punctures; admedian line weakly impressed; pronotal lobe white; tegula yellowish	***S.capoblongus* sp. n**
–	Frontal furrow distinctly impressed; gena with midsize punctures; admedian line distinctly impressed; pronotal lobe ivory; tegula dark brown	***S.japonicus* Tsuneki**
7	Gena finely punctate; mesoscutum with tiny punctures; admedian and parapsidal line weakly impressed	***S.convergensami* Tsuneki**
–	Gena impunctate; mesoscutum with large punctures; admedian and parapsidal line distinctly impressed	***S.shirozuialishanus* Tsuneki**

## Taxonomy

### Family Crabronidae

#### Subfamily Pemphredoninae

##### Genus *Stigmus* Panzer, 1804

**Type species.***Stigmuspendulus* Panzer, 1804, by monotypy.

###### Stigmus capoblongus

Taxon classificationAnimaliaHymenopteraCrabronidae

Bashir & Ma sp. n.

http://zoobank.org/CA9A6376-28E7-42A3-9846-78C3BB171BAE

Figs 1 , 6a

####### Type material.

Holotype: ♂, China: Gansu: Dangxian: Daheba, 35°32'N, 105°17'E, 30.VII.2004, 2003m, No. 200707614, coll. Qiong Wu (ZJU); Paratypes: 3♂, China: Gansu: Dangxian: Daheba, 35°32'N, 105°17'E, 30.VII.2004, 2530m, No. 200707818, 200707830, coll. Min Shi, No. 200707834, coll. Qiong Wu (ZJU); 1♂, China: Shanxi: Liuba: Ziboshan, 38°19'N, 111°28'E, 2004.VIII.3, 1632m, No .200707852, coll. Min Shi (ZJU); 1♂, China: Henan: Funiushan Mount, 33°37'N, 111°43'E, 10.VII.1996, No. 973367, coll. Ping Cai (ZJU).

####### Diagnosis.

Differs from *S.japonicus*Tsuneki (1954) by frontal furrow weakly impressed, inconspicuously; median and upper frons with fine sparse punctures; admedian line weakly impressed; lateral surface of propodeum shiny and smooth anteriorly and medially, distinctly coriaceous mixed with several longitudinal rugae posteriorly. Closely related to *S.quadriceps* Tsuneki but differs by free margin of clypeus with two triangular teeth medially; flagellomere beneath fulvous, above, remaining reddish brown to dark brown; scutellum shiny, with fine sparse punctures; petiole subquadrate (non-cylindrical); pronotal collar with strong carinae anteriorly, lateral carina lacking, without antero-lateral corner.

####### Description.

Male (Figs 1, 6a):

***Measurements.*** BL: 5–5.5 mm; HW : HLD : HLF = 76 : 43 : 57; HW : EWd : EW : TW : EL = 76 : 23 : 26 : 20 : 46; length of scape : length of pedicel : length of flagellomere I : width of flagellomere I : length of flagellomere II : width of flagellomere II = 21 : 8 : 9 : 3 : 8 : 3; PL : PW : LTI : WTI = 38 : 8 : 36 : 40.

***Colour pattern.*** Clypeus with reddish brown to dark brown band subapically; mandible yellowish except reddish brown apically; palpi ivory; scape beneath ivory, above fulvous; pedicel fulvous; flagellomere beneath fulvous, above I fulvous, remaining reddish brown to dark brown; pronotal lobe white; tegula yellowish; forewing veins brown; fore leg: yellowish to fulvous except outer margin of femur somewhat brown, coxa dark brown largely; mid leg: yellowish to fulvous except outer margin of femur somewhat brown, coxa dark brown largely; hind leg: coxa apically, trochanter, base and apex of femur, tibia largely, tarsi yellowish to fulvous, remainder dark brown; petiole black; gaster dark brown, gastral sterna II–VII posteriorly bright yellow; setae on clypeus silvery and mandible yellow.

**Figure 1.**
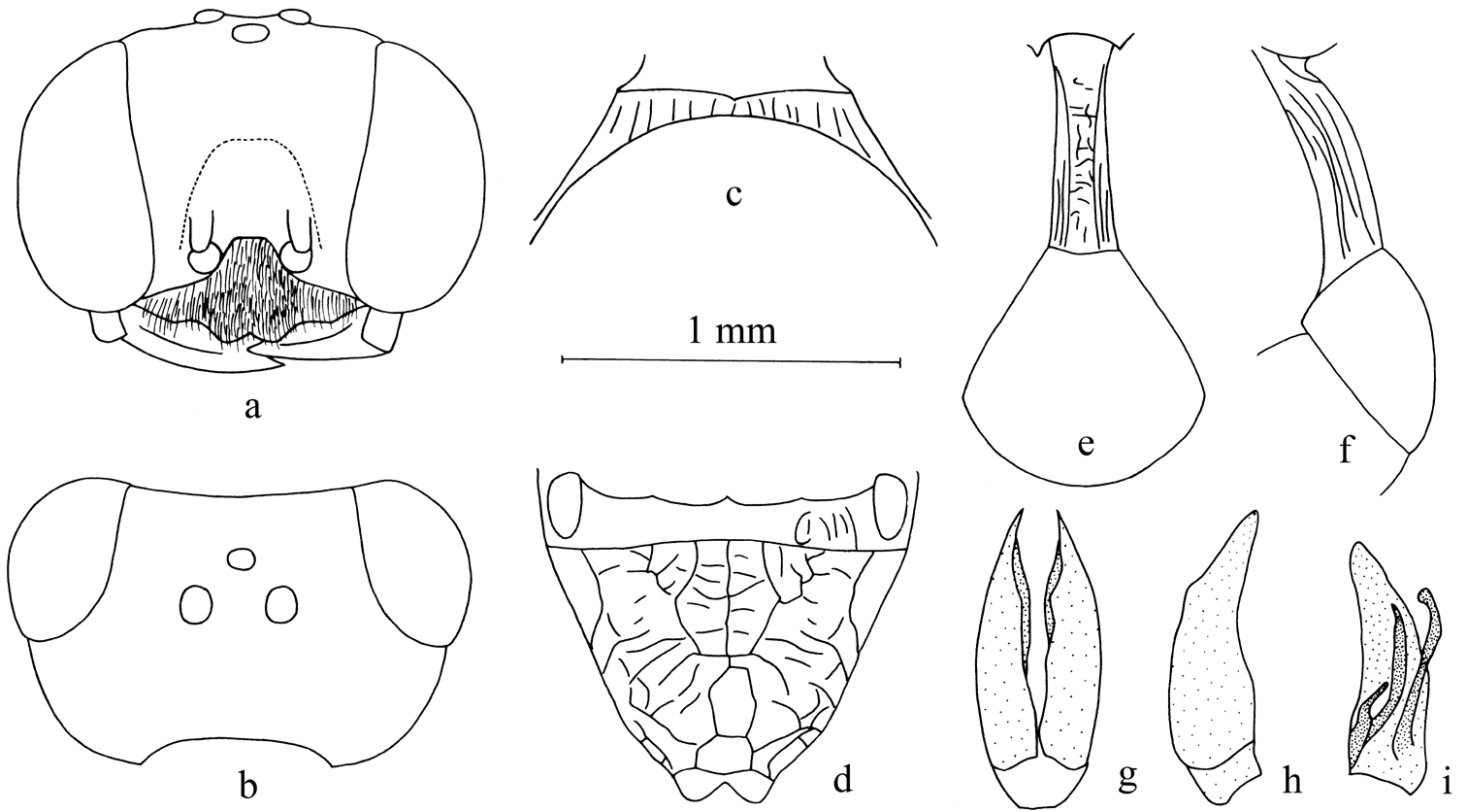
*Stigmuscapoblongus* Bashir & Ma, sp. n. (male). **a** Frontal view of head **b** dorsal view of head **c** dorsal view of collar **d** dorsal view of propodeum **e** dorsal view of petiole and gastral tergum I **f** lateral view of petiole and gastral tergum I **g** dorsal view of male genitalia **h** lateral view of male genitalia **i** ventral view of male genitalia. Scale bars: 1 mm (**a–f**); 1.24 mm (**g–i**).

***Head.*** Mandible bidentate apically (Fig. 1a). Clypeus nearly flat, with dense tiny punctures, setae on clypeus dense, short; free margin of clypeus slightly produced and with two triangular teeth medially, slightly reflected (Fig. 1a). Scapal hollow half mat, coriaceous, somewhat shallow, provided with a vestigial minute tubercle medially, not spined. Frontal furrow very fine and weakly impressed, inconspicuously, sometimes lacking. Median and upper frons shiny, with fine sparse punctures, gently convex. Ocellar triangle area flat, shiny, impunctate, area near eyes with dense, short, impressed lines, opaque area smaller than hind ocellus. Basal half of vertex shiny, with sparse fine punctures, posterior area half mat, with inconspicuous microsculpture and fine sparse punctures (Fig. 1b). Gena shiny, with several fine punctures dorsally, ventral gena shiny and smooth. Head from above with temples slightly convergent posteriorly. Occipital carina incomplete, not ending in hypostomal carina, suddenly ended at the posterior ridge of stomal hollow, not tooth, much narrowed, no crenulate; inner and outer orbital furrows lacking; flagellomeres without tyloids, normal.

***Mesosoma.*** Pronotal collar with strong carinae anteriorly, lateral carina lacking, without antero-lateral corner (Fig. 1c). Mesoscutum shiny, with several fine punctures, anterior area with dense large punctures medially; admedian line weakly impressed, extending to half of scutum. Prescutal sutures deeply grooved and crenulate, reaching one third of scutum. Parapsidal line distinct. Scutellum shiny, with fine sparse punctures. Metanotum slenderly coriaceous. Mesopleuron shiny, with tiny, sparse or dense punctures, posterior mesopleuron with sparse, short, sturdy, longitudinal rugae, episcrobal area with dense, fairly slender, longitudinal rugae, scrobal suture, omaulus and hypersternaulus broadened, distinctly crenate, scrobal suture complete. Propodeal enclosure U-shaped medially, and with a sturdy, longitudinal median carina and sparse transvers rugae, with several sturdy, oblique longitudinal rugae laterally (Fig. 1d). Posterior surface of propodeum with irregular rugae, groove inconspicuous. Lateral surface of propodeum shiny and smooth anteriorly and medially, distinctly coriaceous mixed with several longitudinal rugae posteriorly.

***Legs.*** Normal, outer surface of hind tibia with three long, slender, fulvous to dark brown, spines.

***Wings.*** Forewing venation typical for genus *Stigmus*, hindwing media diverging beyond cu-a.

***Metasoma.*** Dorsal surface of petiole subquadrate (cross section), slightly convex and widened toward apex slightly, and with two sturdy, longitudinal median carinae, area between carinae with dense, sturdy, irregular rugae, median and posterior areas with two sturdy, longitudinal, lateral carinae on each side (Fig. 1e). Lateral surface of petiole with a few strong longitudinal carinae (Fig. 1f). Ventral surface of petiole with 4 sturdy, short, longitudinal carinae posteriorly. Gaster segments shiny, nearly impunctate. Male genitalia (Fig. 1g–i).

***Female.*** Unknown.

####### Distribution.

China (Gansu, Shanxi).

####### Etymology.

The specific name, *capoblongus*, is derived from the Latin *cap*- (= head) and the Latin word *oblongus* (= oblong), referring to the oblong head.

###### Stigmus denticorneus

Taxon classificationAnimaliaHymenopteraCrabronidae

Bashir & Ma sp. n.

http://zoobank.org/D79E43E1-99E2-4D66-8B65-B2E714AE7C58

Figs 2 , 6b, c

####### Type material.

Holotype: ♀, China: Gansu: Dangxian: Daheba, 35°32'N, 105°17'E, 30.VII.2004, 2530m, No. 200707781, coll. Qiong Wu (ZJU); Paratypes: 1♀7♂, China: Gansu: Dangxian: Daheba, 35°32'N, 105°17'E, 30.VII.2004, 2530m, ♀, No. 200707788, 7♂, No. 200707785, 200707812, 200707771, 200707795, 200707816, 200707814, 200707829, coll. Qiong Wu, Min Shi (ZJU).

####### Diagnosis.

Distinguished from *S.japonicus* by combination of characters: in female, free margin of clypeus slightly produced and with two distinct cornuted teeth medially, deeply emarginated in the middle; scrobal suture inconspicuous, just with several longitudinal rugae; lateral surface of petiole with two strong longitudinal carinae; admedian and parapsidal line weakly impressed; posterior surface of propodeum with a shallow narrow median groove, shiny, remaining with contiguous punctures and sparse irregular oblique longitudinal rugae. Closely related to *S.quadriceps* except antenna dark brown; forewing veins brown; gena with sparse midsize to large punctures dorsally; in male, frontal furrow distinctly impressed on upper frons; free margin of clypeus slightly produced and nearly truncate medially; anterior area of pronotal collar narrowly emarginated in middle, antero-lateral corner lacking; petiole subquadrate (non-cylindrical), slightly convex, not longer than 1^st^ abdominal tergite.

####### Description.

**Figure 2.**
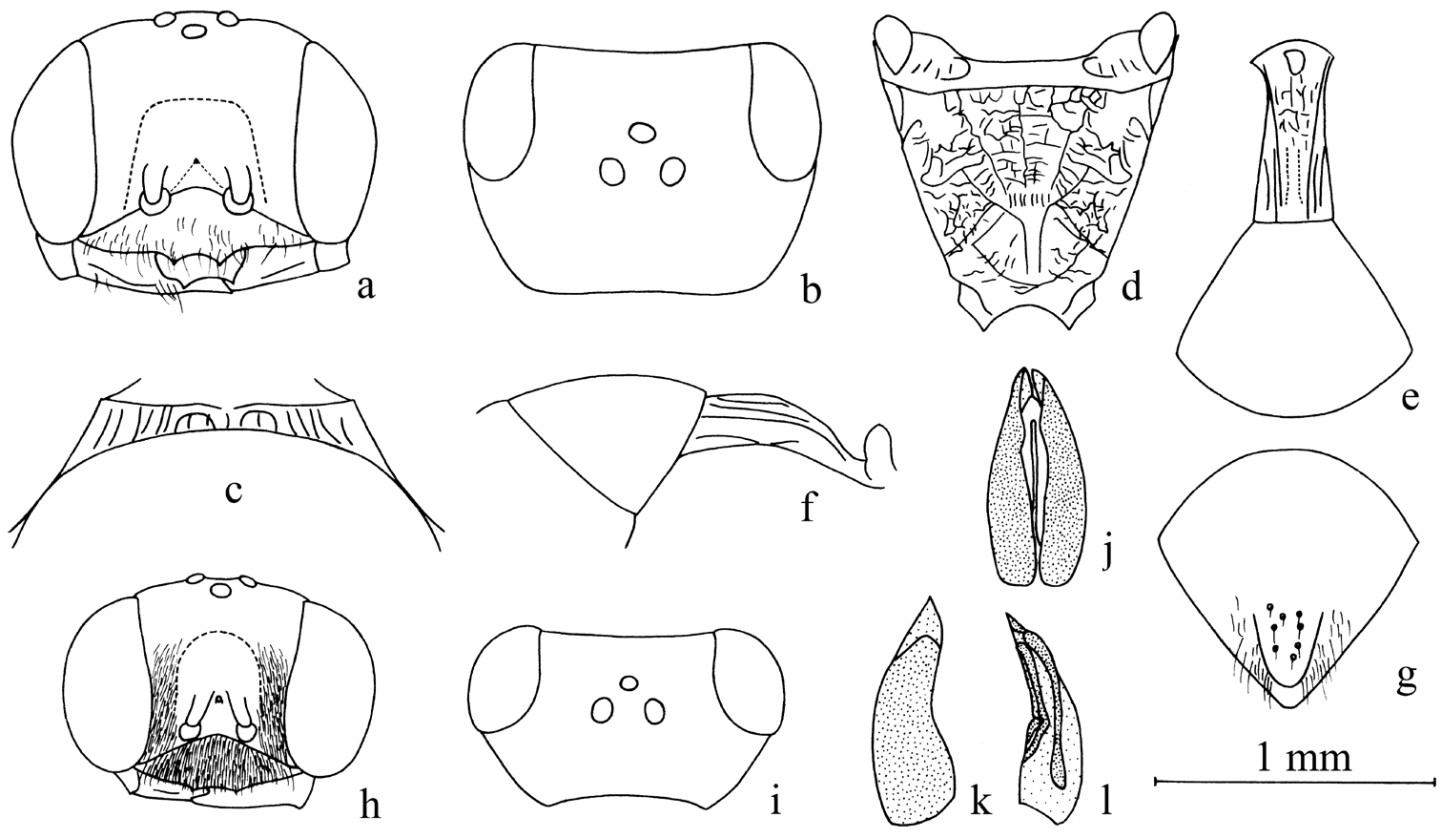
*Stigmusdenticorneus* Bashir & Ma, sp. n. (**a–g** female **h–l** male). **a, h** Frontal view of head **b, i** dorsal view of head **c** dorsal view of collar **d** dorsal view of propodeum **e** dorsal view of petiole and gastral tergum I **f** lateral view of petiole and gastral tergum I **g** dorsal view of pygidial plate **j** dorsal view of male genitalia **k** lateral view of male genitalia **l** ventral view of male genitalia. Scale bars: 1 mm (**a, b, d–f, h, i**); 1.63 mm (**c, g, j–l**).

Female (Figs 2a–g, 6b):

***Measurements.*** ♀ BL: 5 mm; HW : HLD : HLF = 68 : 42 : 52; HW : EWd : EW : TW : EL = 68 : 15 : 20 : 21 : 42; length of scape : length of pedicel : length of flagellomere I : width of flagellomere I : length of flagellomere II : width of flagellomere II = 20 : 8 : 6 : 4 : 6 : 4; PL : PW : LTI : WTI = 37 : 9 : 32 : 38. ♂, BL: 3.8–4.2 mm; HW : HLD : HLF = 62 : 36 : 48; HW : EWd : EW : TW : EL = 62 : 16 : 21 : 14 : 38; length of scape : length of pedicel : length of flagellomere I : width of flagellomere I : length of flagellomere II : width of flagellomere II = 15 : 7 : 6 : 3 : 7 : 3.5; PL : PW : LTI : WTI = 34 : 10 : 28 : 28.

***Colour pattern.*** Clypeus with reddish brown to dark brown band subapically; mandible yellowish except reddish brown apically; labrum dark brown; palpi fulvous; antenna beneath fulvous and dark brown; pronotal lobe ivory; tegula fulvous; forewing veins brown; fore and mid legs: base and apex of femur, tibia, tarsi fulvous, trochanter and remaining femur dark brown; hind leg basal one fourth of tibia and tarsus fulvous, remaining tibia dark brown; petiole black; gaster dark brown; setae on clypeus and mandible yellow.

***Head.*** Mandible tridentate apically, median tooth larger (Fig. 2a). Labrum pentagonal, apex deeply emarginated (Fig. 2a). Clypeus shiny, slightly convex, with sparse midsize punctures, setae on clypeus sparse, long; free margin of clypeus slightly produced and with two distinct cornuted teeth medially, slightly reflected (Fig. 2a). Scapal hollow half mat, coriaceous, somewhat shallow, provided with a vestigial minute tubercle medianly, not spined. Frontal furrow very fine and weakly impressed, inconspicuous. Median and upper frons shiny, with fine sparse punctures, gently convex. Ocellar triangle area flat, shiny, impunctate, near eyes area with dense, short, impressed lines, opaque area smaller than hind ocellus. Vertex behind ocelli half mat, with slender microsculpture, and fine sparse punctures. Gena shiny, with sparse midsize to large punctures dorsally (Fig. 2b), ventral gena shiny, smooth. Head from above with temples rarely convergent posteriorly, subquadrate. Occipital carina incomplete, not ending in hypostomal carina, suddenly ended at the posterior ridge of stomal hollow, not tooth, much narrowed, no crenulate; inner and outer orbital furrows lacking.

***Mesosoma.*** Anterior area of pronotal collar with sturdy carinae (incomplete), narrowly emarginated in middle, lateral carina lacking, antero-lateral corner lacking (Fig. 2c). Mesoscutum half mat, anterior area with large dense punctures and slender coriaceous, remaining with sparse, midsize to large punctures. Admedian line weakly impressed, extending to half of scutum. Prescutal sutures deeply grooved, reaching one third of scutum. Parapsidal line weakly impressed. Scutellum shiny, with fine sparse, punctures; metanotum slenderly coriaceous. Mesopleuron shiny, with sparse, midsize punctures, episcrobal area with dense longitudinal rugae, omaulus and hypersternaulus broadened, distinctly crenate, scrobal suture inconspicuous, just with several longitudinal rugae. Propodeal enclosure elongate, U-shaped medially, and with a sturdy longitudinal median rugae and sparse transvers rugae, lateral area with contiguous, irregular rugae and punctures mixed with several, sturdy, oblique longitudinal rugae laterally (Fig. 2d). Posterior surface of propodeum with a shallow narrow median groove, shiny, remaining with contiguous punctures and sparse, irregular, oblique longitudinal rugae (Fig. 2d). Lateral surface of propodeum with slender, contiguous mixed with several sturdy, oblique longitudinal rugae.

***Legs.*** Normal, outer surface of hind tibia with three long, slender, fulvous to dark brown, spines.

***Wings.*** Forewing venation typical for genus *Stigmus*, hindwing media diverging beyond cu-a.

***Metasoma.*** Dorsal surface of petiole subquadrate (cross section), slightly convex and widened toward apex slightly, with two strong longitudinal carinae, and irregular, strong rugae anteriorly and medially, lateral area with 2 strong longitudinal carinae posteriorly on each side (Fig. 2e). Lateral surface of petiole with a few strong longitudinal carinae (Fig. 2f). Ventral surface of petiole with a few strong, longitudinal carinae medially and posteriorly. Gastral segments shiny, nearly impunctate, gastral sternum VI with fine, coarse punctures, half mat. Pygidial area shiny, broadly U-shaped, apex rounded, with 2 lines, large punctures and setae medially (Fig. 2g).

***Male*** (Figs 2h–l, 6c). Almost same to female except mandible ivory with reddish brown apically; palpi ivory; flagellomere reddish brown to dark brown; pronotal lobe white; fore and mid legs: trochanter, base and apex of femur, tibia, tarsi yellowish to fulvous, remaining dark brown; setae on clypeus dense, silvery, short. Mandible bidentate apically (Fig. 2h); clypeus near flat, with dense tiny punctures; free margin of clypeus slightly produced and nearly truncate medially, moderately reflected apically (Fig. 2h); frontal furrow distinctly impressed on upper frons; median and upper frons shiny, with several midsize to large punctures, strongly convex; ocellar triangle area slightly convex, shiny, impunctate, near eyes area with dense, short, impressed lines, opaque area large (Fig. 2i); vertex behind ocelli half mat, slenderly coriaceous, with several shallow, midsize punctures; gena shiny, inconspicuous coriaceous, with several fine to midsize punctures dorsally; head from above with temples somewhat roundly convergent posteriorly (Fig. 2i); occipital carina incomplete, distinctly crenulate; flagellomeres without tyloids, normal. Male genitalia (Fig. 2j–l).

####### Distribution.

China (Gansu).

####### Etymology.

The specific epithetic, is derived from the Latin *dent*- (= tooth) and the Latin word *corneus* (= cornuted), referring to the free margin of clypeus with two distinct cornuted teeth medially.

###### Stigmus fronticoncavus

Taxon classificationAnimaliaHymenopteraCrabronidae

Bashir & Ma sp. n.

http://zoobank.org/C03B9D82-3729-4CD5-8D49-2A77FA164F06

Figs 3 , 6d

####### Type material.

Holotype ♀, China: Yunnan: Ruili: Mengxiu, 24°05'N, 97°47'E, 2.V.1981, coll. Fasheng Li (CAU).

####### Diagnosis.

Similar to *S.murotai* (Tsuneki, 1977) but differ by clypeus impunctate; free margin of clypeus not produced, with two small teeth medially, nearly truncate apically; labrum five lobed; ventral surface of petiole smooth, without carina; gena impunctate; parapsidal line weakly impressed; pygidial area broadened triangular shaped. *S.murotai* has the following characters: clypeus with sparse, fine punctures; free margin of clypeus narrowly produced, with two triangular teeth medially, slightly emarginated in middle; labrum trapeziform; ventral surface of petiole with dense, sturdy, short, longitudinal carinae posteriorly; gena with fine punctures; parapsidal line distinct; pygidial area broadened U-shaped.

####### Description.

**Figure 3.**
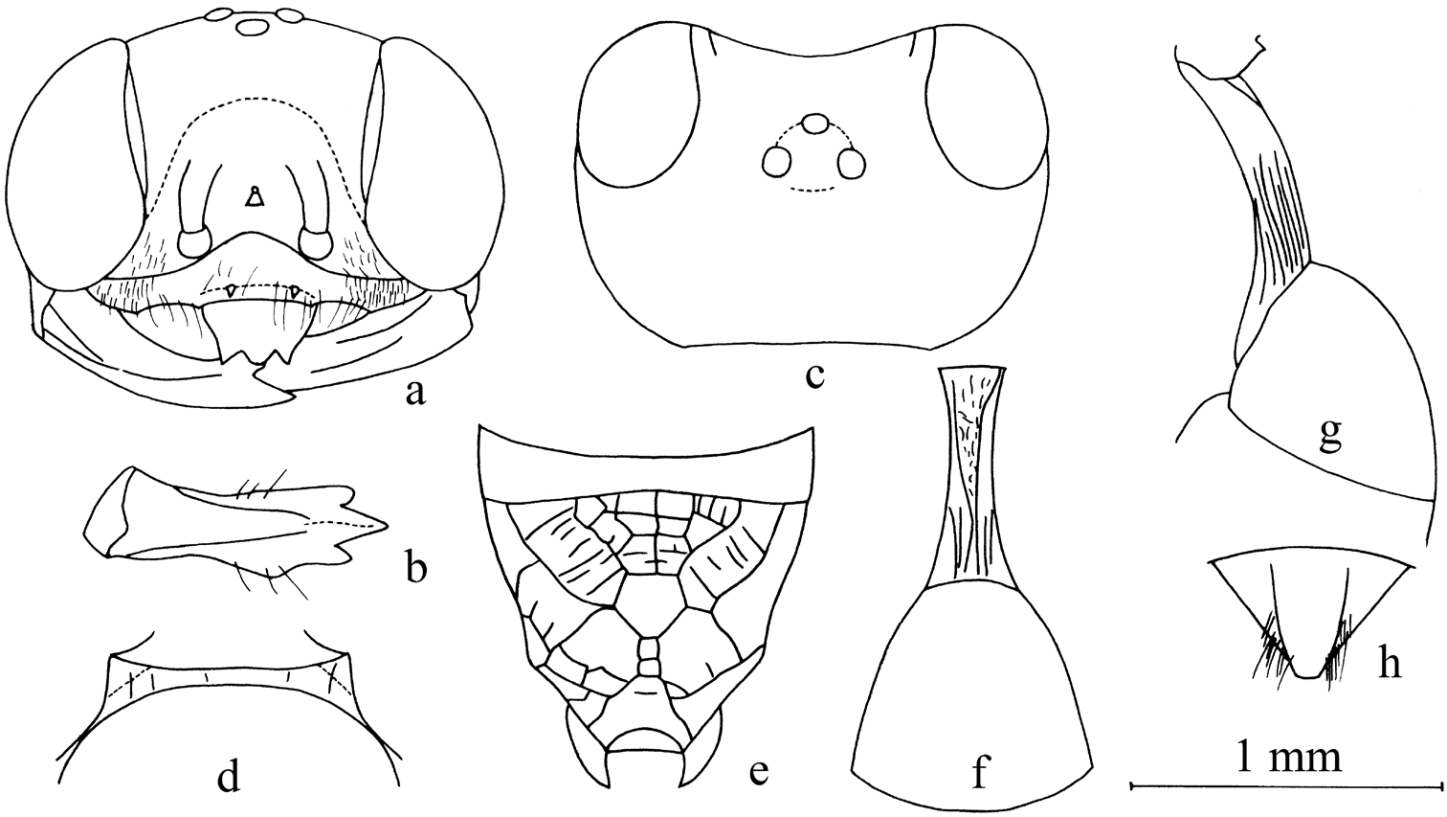
*Stigmusfronticoncavus* Bashir & Ma, sp. n. (female). **a** Frontal view of head **b** ventral view of mandible **c** dorsal view of head **d** dorsal view of collar **e** dorsal view of propodeum **f** dorsal view of petiole and gastral tergum I **g** lateral view of petiole and gastral tergum I **h** dorsal view of pygidial plate. Scale bars: 1 mm (**a–h**).

Female (Figs 3, 6d):

***Measurements.*** BL: 5.3 mm; HW : HLD : HLF = 81 : 53 : 53; HW : EWd : EW : TW : EL = 81 : 22 : 23 : 28 : 50; length of scape : length of pedicel : length of flagellomere I : width of flagellomere I : length of flagellomere II : width of flagellomere II = 28 : 8 : 8 : 3.5 : 9 : 4; PL : PW : LTI : WTI = 34 : 8 : 40 : 43.

***Colour pattern.*** Clypeus dark brown apically; mandible yellowish except reddish brown apically; labrum, palpi, scape, tegula and pedicel fulvous; flagellomere I–VI segments fulvous, VII-X reddish brown to dark brown; pronotal lobe yellowish; forewing veins brown; legs fulvous except coxa dark brown basally; petiole black; metasoma black, last segment dark brown; setae on clypeus and mandible golden.

***Head.*** Mandible tridentate apically (Fig. 3a), median tooth larger, outer margin of mandible with a broad triangular tooth nearly apical area (Fig. 3b). Labrum with five lobes, apex with two lateral teeth and round teeth medially (Fig. 3a). Clypeus shiny, impunctate, fairly deeply impressed, setae on clypeus sparse, long; free margin of clypeus not produced, nearly truncate apically, and with two small teeth medially and two blunt teeth laterally, median teeth slightly reflected (Fig. 3a). Scapal hollow shiny, fairly deep and broad, not well outlined, provided with a small round tubercle medially, not spined. Frontal furrow lacking. Median and upper frons shiny, impunctate. Ocellar triangle area flat, shiny, impunctate, near eyes area with 3 or 4 short impressed lines, opaque area small. Vertex behind ocelli shiny, impunctate; gena shiny, smooth and impunctate (Fig. 3c). Head from above with temples somewhat roundly convergent posteriorly. Occipital carina incomplete, not ending in hypostomal carina, suddenly ended at the posterior ridge of stomal hollow, forming a blunt tooth, much narrowed, not crenulate. Inner orbital furrow broad, shiny, with inner marginal carina distinct; outer orbital furrow lacking.

***Mesosoma.*** Anterior and lateral areas of pronotal collar with strong carinae, forming blunt angle at antero-lateral corner (Fig. 3d). Mesoscutum shiny, with tiny sparse punctures. Admedian line weakly impressed, extending to half of scutum. Prescutal sutures shallowly grooved and crenulate, reaching only anterior of scutum; parapsidal line weakly impressed. Scutellum shiny, with fine sparse punctures. Metanotum slenderly rugulose. Mesopleuron with dense sparse punctures, posterior mesopleuron shiny, with several short, slender, longitudinal rugae, episcrobal area with sparse, irregular, longitudinal rugae, scrobal suture, omaulus and hypersternaulus broadened, distinctly crenate, scrobal suture complete. Propodeal enclosure triangular medially (Fig. 3e), with a sturdy longitudinal median carina and sparse transvers rugae, with sparse sturdy oblique longitudinal rugae laterally; posterior surface of propodeum with sparse irregular rugae, forming several smooth areas (Fig. 3e); lateral surface of propodeum with sparse, sturdy, oblique longitudinal rugae.

***Legs.*** Normal, outer surface of hind tibia with three long, slender, fulvous to dark brown, spines.

***Wings.*** Forewing venation typical for genus *Stigmus*, hindwing media diverging before cu-a.

***Metasoma.*** Dorsal surface of petiole subquadrate (cross section), slightly convex and widened toward apex distinctly, basal half of petiole with two sturdy lateral carinae and dense irregular rugae, apex with dense, sturdy, longitudinal carinae posteriorly (Fig. 3f); lateral surface of petiole with a few strong, longitudinal carinae medially and posteriorly (Fig. 3g); ventral surface of petiole shiny, smooth, without carina. Metasomal segments shiny, nearly impunctate, gastral sternum VI with fine or coarse punctures, half mat; pygidial area shiny, broadly triangular, smooth (Fig. 3h).

***Male.*** Unknown.

####### Distribution.

China (Yunnan).

####### Etymology.

The name, *fronticoncavus*, is derived from the Latin *front*- (= frons) and the Latin word *concavus* (= concave), referring to the hollow, deep and broad scapal.

###### Stigmus interruptus

Taxon classificationAnimaliaHymenopteraCrabronidae

Bashir & Ma sp. n.

http://zoobank.org/636548BE-E8B7-47D5-A59F-D71CBCC4DB21

Figs 4 , 6e

####### Type material.

Holotype ♀, China: Tibet: Linzhi, 29°42'N, 87°21'E, 20.VIII.2003, No. 20035170, coll. Dejimeiduo (ZJU); Paratypes: 2♀, same data as Holotype except No. 20035185, 20034328; 1♀, China: Tibet: Sejilashan Mount, 29°59'N, 94°54'E, 1.IX.2002, No. 20032992, coll. Naiquan Lin (ZJU).

####### Diagnosis.

Distinguished from closely related species *S.japonicus* by pronotal lobe white; median and upper frons with midsize punctures; vertex behind ocelli with midsize punctures; pygidial area half mat, apex truncate; lateral surface of petiole with two strong longitudinal carinae; mesoscutum with sparse large punctures; posterior surface of propodeum with a shallow somewhat narrow median groove, remaining with contiguous punctures and several oblique longitudinal rugae. *Stigmusjaponicus* has following characters: pronotal lobe ivory; median and upper frons with fine punctures; vertex behind ocelli impunctate; pygidial area shiny, apex round; lateral surface of petiole with a few strong longitudinal carinae; mesoscutum with fine sparse punctures; posterior surface of propodeum with irregular rugae, groove inconspicuous.

####### Description.

**Figure 4.**
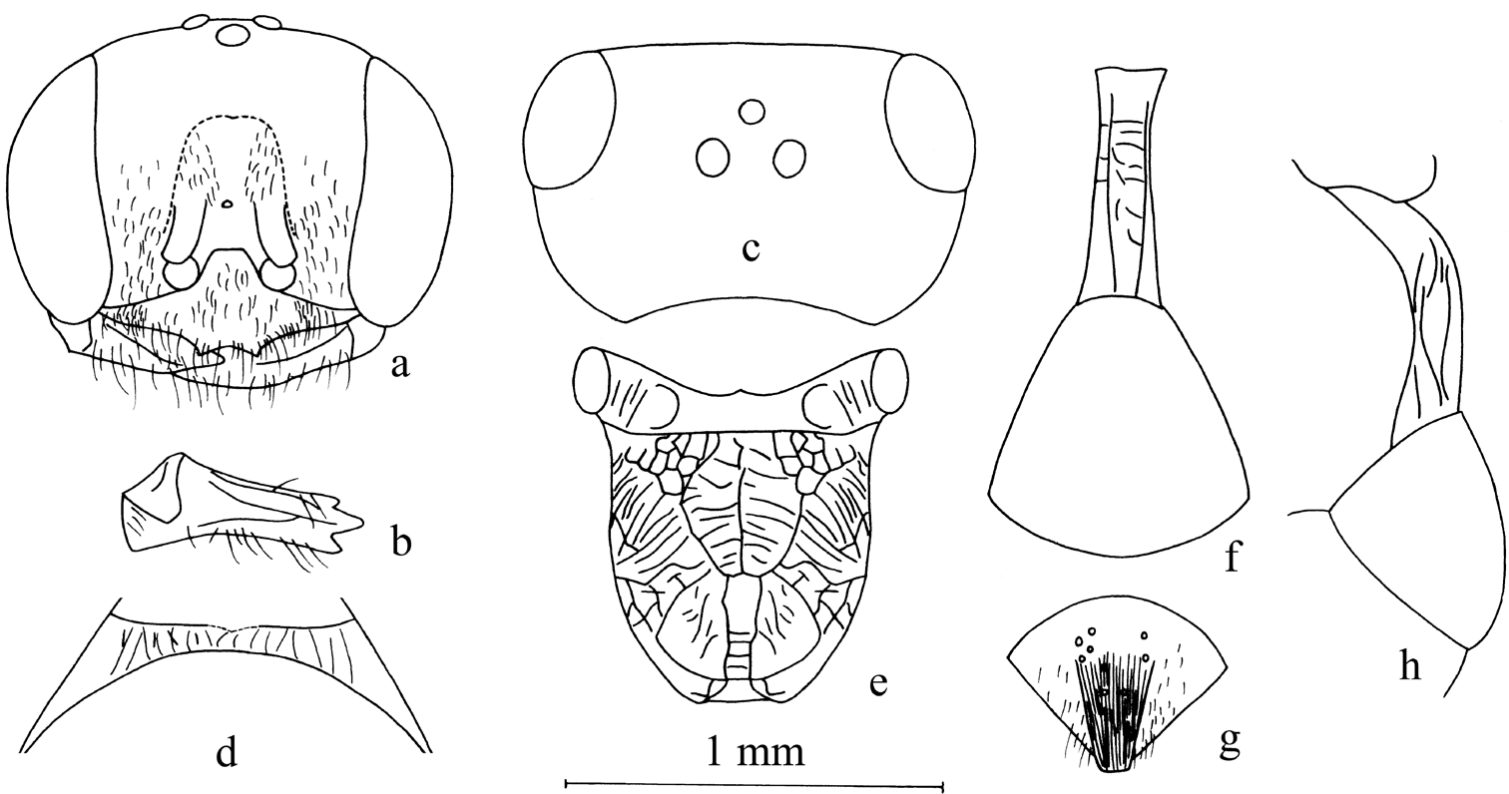
*Stigmusinterruptus* Bashir & Ma, sp. n. (female). **a** Frontal view of head **b** ventral view of mandible **c** dorsal view of head **d** dorsal view of collar **e** dorsal view of propodeum **f** dorsal view of petiole and gastral tergum I **g** dorsal view of pygidial plate **h** lateral view of petiole and gastral tergum I. Scale bars: 1 mm (**a, c, e, f, h**); 0.95 mm (**b**); 1.24 mm (**d, g**).

Female (Figs 4, 6e):

***Measurements.*** BL: 4.3–4.8 mm; HW : HLD : HLF = 65 : 40 : 55; HW : EWd : EW : TW : EL = 65 : 14 : 18 : 21 : 42; length of scape : length of pedicel : length of flagellomere I : width of flagellomere I : length of flagellomere II : width of flagellomere II = 20 : 7 : 8 : 4 : 8 : 4.5; PL : PW : LTI : WTI = 34 : 9 : 34 : 36.

***Colour pattern.*** Clypeus with reddish brown to dark brown band subapically; mandible ivory except reddish brown apically; palpi yellowish; scape beneath ivory, above dark brown largely; pedicel beneath fulvous, dark brown above; flagellomere dark brown to black; pronotal lobe white; tegula fulvous; forewing veins dark brown; fore and mid tibia, tarsi, femur (base and apex), trochanter, hind coxa, basal one third of hind tibia (remaining tibia dark brown) fulvous; petiole black; metasoma black except last segment reddish brown apically; setae on clypeus and mandible silvery.

***Head.*** Mandible tridentate apically (Fig. 4a), median tooth larger (Fig. 4b). Clypeus shiny, flat, with sparse, midsize punctures, setae on clypeus sparse, long; free margin of clypeus narrowly produced and with two triangular teeth medially, slightly reflected (Fig. 4a). Scapal hollow shiny, somewhat shallow, broadened, not well outlined, without tubercle medially. Frontal furrow very fine and weakly impressed, inconspicuous. Median and upper frons shiny, with midsize sparse punctures, gently convex. Ocellar triangle area flat, shiny, impunctate, near eyes area with dense, short, impressed lines, opaque area large. Vertex behind ocelli shiny, with several midsize punctures (Fig. 4c). Gena shiny, with sparse, midsize to large punctures dorsally, ventral gena shiny and smooth. Head from above with temples somewhat roundly convergent posteriorly. Occipital carina incomplete, not ending in hypostomal carina, suddenly ended at the posterior ridge of stomal hollow, not tooth, much narrowed, no crenulate; inner and outer orbital furrows lacking.

***Mesosoma.*** Anterior area of pronotal collar with sturdy carinae, incomplete, narrowly emarginated in middle, lateral carina lacking, without antero-lateral angle (Fig. 4d). Mesoscutum half mat, with sparse large punctures, anterior and posterior areas with dense, longitudinal micro-sculptures. Admedian line distinct, extending to half of mesoscutum. Prescutal sutures deeply grooved and crenulate, reaching half of mesoscutum. Parapsidal line distinct. Scutellum shiny, with fine sparse punctures. Metanotum slenderly coriaceous. Mesopleuron shiny, smooth, episcrobal area with dense, slender, longitudinal rugae, scrobal suture, omaulus and hypersternaulus narrowed, distinctly crenate, scrobal suture complete. Propodeal enclosure elongate, U-shaped medially, and with a sturdy longitudinal median rugae and sparse irregular transvers rugae (Fig. 4e), lateral area with contiguous irregular rugae mixed with sparse, slender, oblique longitudinal rugae, laterally. Posterior surface of propodeum with somewhat narrow median groove, shiny, with several transverse rugae, remaining with contiguous punctures and several oblique longitudinal rugae (Fig. 4e); lateral surface of propodeum with contiguous punctures and slender mixed with several sturdy, oblique longitudinal rugae.

***Legs.*** Normal, outer surface of hind tibia with three long, slender, fulvous to dark brown, spines.

***Wings.*** Forewing venation typical for genus *Stigmus*, hindwing media diverging beyond cu-a.

***Metasoma.*** Dorsal surface of petiole subquadrate (cross section), strongly convex and widened toward apex slightly, and with two sturdy, longitudinal median carinae, and with irregular strong rugae anteriorly and medially (Fig. 4f); lateral surface of petiole with a few strong longitudinal carinae medianly and posteriorly (Fig. 4h); ventral surface of petiole with a few strong longitudinal carinae medianly and posteriorly. Metasomal segments shiny, nearly impunctate, gastral sternum VI with fine or coarse punctures, half mat; pygidial area half mat, broadly U-shaped, apex truncate, with longitudinal micro-sculptures, basal area with several large punctures (Fig. 4g).

***Male.*** Unknown.

####### Distribution.

China (Tibet).

####### Etymology.

The name, *interruptus*, is derived from the Latin word *interruptus* (= interrupt), referring to the anterior area of the pronotal collar with sturdy carinae, incomplete, narrowly emarginate in the middle.

###### Stigmus lobomelanicus

Taxon classificationAnimaliaHymenopteraCrabronidae

Bashir & Ma sp. n.

http://zoobank.org/5FB42D4C-37DE-4C6E-A152-3DDE420A0BD8

Figs 5 , 6f, g

####### Type material.

Holotype ♀, China: Yunnan: Xishuangbanna: Jinghong: Yexianggu, 22°09'N, 100°52'E, 23.IX.2006, coll. Hesheng Wang (YNAU); Paratypes: 1♀, China: Yunnan: Ruili: Nanjingli, 24°05'N, 97°47'E, 5.V.1981, coll. Fasheng Li (CAU); 1♂, China: Yunnan: Ruili, 23°59'N, 97°37'E, 2.V.1981, No. 812489, coll. Junhua He (CAU); 1♂, China: Yunnan: Mengla: Wangtianshu Forest Park, 22°01'N, 100°47'E, 2.V.2005, coll. Peng Wang (YNAU); 1♀1♂, China: Guizhou: Luodian, 25°13'N, 105°50'E, 2–5.VI.1981, coll. Fasheng Li (CAU); 1♀, China: Yunnan: Menghai, 22°27'N, 98°20'E, 17.V.1981, coll. Fasheng Li (CAU).

####### Diagnosis.

Differs from *S.pendulus* by free margin of clypeus strongly produced and truncate medially, frontal furrow lacking, gena with large dense punctures, scutellum with several large punctures, pygidial area broadly triangular, with dense, slender, longitudinal striations; from *S.munakatai* Tsuneki it differs by setae on clypeus and mandible golden, upper frons with midsize to large punctures, inner orbital furrow broad, pronotal collar without antero-lateral angle, scutellum with large punctures, propodeum strongly reticulate, lateral surface with longitudinal rugae, pygidial area broadly triangular; in male, free margin of clypeus truncate medially, mandible reddish brown with black basally.

####### Description.

**Figure 5.**
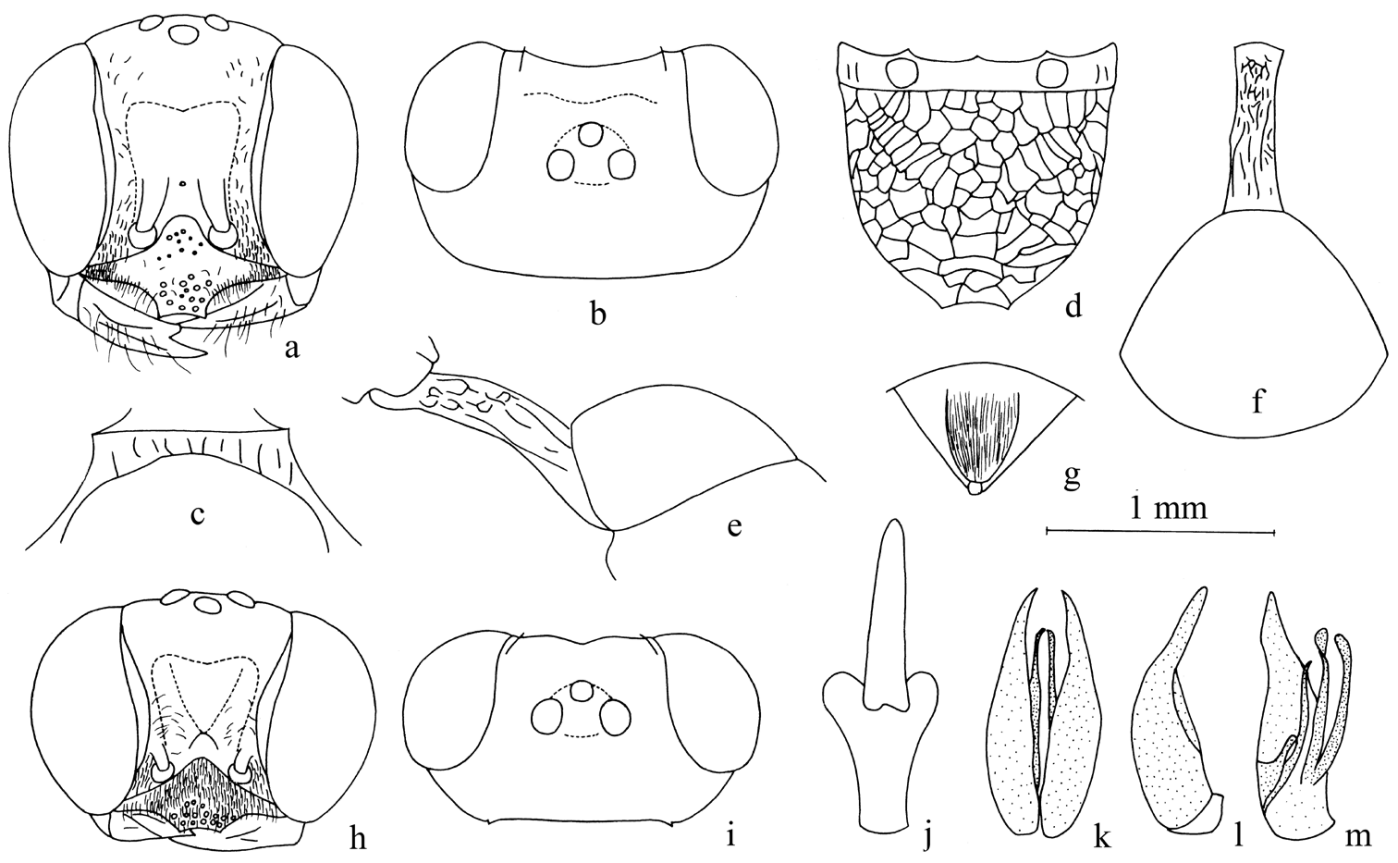
*Stigmuslobomelanicus* Bashir & Ma, sp. n. (**a–g** female **h–m** male). **a, h** Frontal view of head **b, i** dorsal view of head **c** dorsal view of collar **d** dorsal view of propodeum **e** lateral view of petiole and gastral tergum I **f** dorsal view of petiole and gastral tergum I **g** dorsal view of pygidial plate **j** ventral view of gastral tergum VIII **k** dorsal view of male genitalia **l** lateral view of male genitalia **m** ventral view of male genitalia. Scale bars: 1 mm (**a–f, h, i**); 1.63 mm (**g, j–m**).

**Figure 6.**
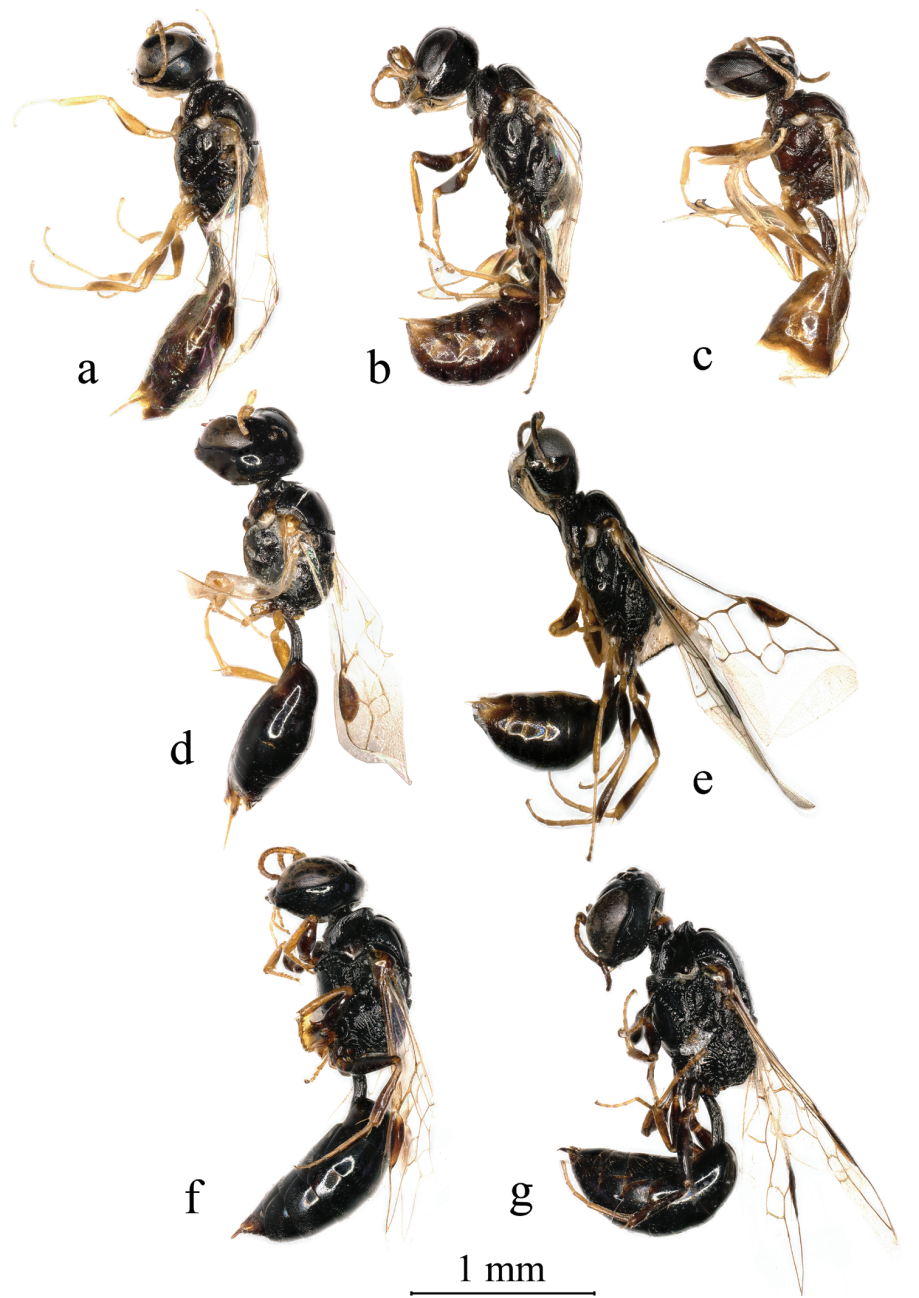
**a***Stigmuscapoblongus* Bashir & Ma, sp. n. (male) **b, c***Stigmusdenticorneus* Bashir & Ma, sp. n. (**b** female **c** male) **d***Stigmusfronticoncavus* Bashir & Ma, sp. n. (female) **e***Stigmusinterruptus* Bashir & Ma, sp. n. (female) **f, g***Stigmuslobomelanicus* Bashir & Ma, sp. n. (**f** female **g** male) **a–g** lateral view, Scale bars: 1 mm (**a–g**).

Female (Figs 5a–g, 6f):

***Measurements.*** ♀ BL: 5.2–6.1 mm; HW : HLD : HLF = 84 : 45 : 70; HW : EWd : EW : TW : EL = 84 : 23 : 28 : 23 : 58; length of scape : length of pedicel : length of flagellomere I : width of flagellomere I : length of flagellomere II : width of flagellomere II = 25 : 8 : 7 : 4 : 7 : 5; PL : PW : LTI : WTI = 35 : 10 : 42 : 58. ♂, BL: 5.2–5.5 mm; HW : HLD : HLF = 81 : 41 : 63; HW : EWd : EW : TW : EL = 81 : 25 : 29 : 15 : 55; length of scape : length of pedicel : length of flagellomere I : width of flagellomere I : length of flagellomere II : width of flagellomere II = 20 : 8 : 6 : 3.5 : 6 : 4; PL : PW : LTI : WTI = 35 : 10 : 42 : 50.

***Colour pattern.*** Clypeus black; mandible reddish brown to dark brown except black basally and apically; palpi dark brown; scape, pedicel, tegula and flagellomere dark brown to black; pronotal lobe black; forewing veins fulvous to dark brown; fore and mid legs: tibia, tarsi, femur (apex) reddish brown to dark brown, hind tarsus dark brown; petiole and metasoma black; setae on clypeus and mandible golden.

***Head.*** Mandible tridentate apically, median tooth larger (Fig. 5a). Clypeus shiny, with sparse fine to midsize punctures, apex with a line, large dense punctures, strongly reflected toward apex gradually, setae on clypeus sparse, long; free margin of clypeus strongly produced and truncate medially (Fig. 5a). Scapal hollow half mat, slenderly coriaceous, somewhat shallow, without tubercle medially. Frontal furrow lacking. Median frons half mat, somewhat coriaceous, upper frons with midsize to large, sparse punctures, slightly convex. Ocellar triangle area flat, shiny, impunctate, near eyes area with dense, short, impressed lines, opaque area smaller than hind ocellus. Vertex behind ocelli shiny, with fine sparse punctures, round posteriorly; gena shiny, with sparse midsize to large punctures (Fig. 5b); ventral gena shiny, with large dense punctures mixed with several irregular rugae laterally. Head from above with temples rarely convergent posteriorly, subquadrate. Occipital carina incomplete, not ending in hypostomal carina, extending to nearly base of mandible, not tooth, outer orbital furrow much narrowed, no crenulate, on lower part somewhat broad, coarsely crenulate. Inner orbital furrow broad, shiny, slenderly rugulose; outer orbital furrow lacking.

***Mesosoma*.** Anterior and lateral areas of pronotal collar with strong carinae, without antero-lateral angle (Fig. 5c). Mesoscutum shiny, with midsize to large punctures, anterior area slenderly coriaceous. Admedian line weakly impressed, extending to one third of scutum. Prescutal sutures deeply grooved and crenulate, reaching only anterior of scutum. Parapsidal line weakly impressed. Scutellum mat, coriaceous, with several large punctures. Metanotum distinctly rugulose. Mesopleuron shiny, with sparse large punctures, posterior mesopleuron with sparse, short, sturdy, longitudinal rugae, episcrobal area with dense reticulation, scrobal suture, omaulus and hypersternaulus much broadened, distinctly crenate, scrobal suture complete. Propodeal enclosure triangular medially, and with sturdy irregular reticulation (Fig. 5d). Posterior surface of propodeum with a fairly broadened shallow median groove, and sparse sturdy transverse rugae in groove, remaining sturdy, irregularly reticulate (Fig. 5d). Lateral surface of propodeum with dense, sturdy, oblique longitudinal rugae anteriorly and medially, and irregular reticulation posteriorly.

***Legs.*** Normal, outer surface of hind tibia with three long, slender, fulvous to dark brown, spines.

***Wings.*** Forewing venation typical for genus *Stigmus*, hindwing media diverging before cu-a.

***Metasoma.*** Dorsal surface of petiole subquadrate (cross section), moderately convex and widened toward apex distinctly, and with strong irregular rugae (Fig. 5f); lateral surface of petiole shiny, with several irregular rugae and two strong lateral carinae medially and posteriorly (Fig. 5e); ventral surface of petiole with 4 sturdy, short, longitudinal carinae posteriorly. Metasomal segments shiny, with fine sparse punctures, gastral sternum VI with fine or coarse punctures, half mat; pygidial area shiny, broadly triangular, with dense, slender, longitudinal striations (Fig. 5g).

***Male*** (Figs 5h–m, 6g). Almost same to female except mandible reddish brown with black basally, setae on clypeus dense, silvery and short; mandible bidentate apically (Fig. 5h); clypeus moderately reflected toward apex gradually, with dense fine punctures (Fig. 5h); vertex behind ocelli impunctate (Fig. 5i); gena shiny, inconspicuous coriaceous, with several large punctures dorsally, ventral gena shiny, with sturdy, sparse, irregular rugae laterally; head from above with temples distinctly convergent posteriorly; flagellomeres without tyloids, normal; dorsal surface of petiole subquadrate (cross section), slightly convex and widened toward apex slightly, and with strong irregular rugae; metasomal segments shiny, nearly impunctate. Sternum VIII (Fig. 5j). Male genitalia (Fig. 5k–m).

####### Distribution.

China (Yunnan, Guizhou).

####### Etymology.

The name, *lobomelanicus*, is derived from the Greek *lob*- (= lobe) and the Greek word *melanicus* (= black), referring to pronotal lobe black.

## Supplementary Material

XML Treatment for Stigmus capoblongus

XML Treatment for Stigmus denticorneus

XML Treatment for Stigmus fronticoncavus

XML Treatment for Stigmus interruptus

XML Treatment for Stigmus lobomelanicus
